# Exploring stimulant treatment in ADHD: narratives of young adolescents and their parents

**DOI:** 10.1186/1471-244X-14-110

**Published:** 2014-04-12

**Authors:** Alice Charach, Emanuela Yeung, Tiziana Volpe, Tara Goodale, Susan dosReis

**Affiliations:** 1 Program in Child Health Evaluative Sciences, Research Institute The Hospital for Sick Children, Toronto, Canada; 2 Department of Psychiatry, University of Toronto Faculty of Medicine, Toronto, Canada; 3 Department of Psychology, University of Victoria, Victoria, Canada; 4 Community Health Systems Resource Group, The Hospital for Sick Children, Toronto, Canada; 5 Program in Neurosciences and Mental Health, Research Institute The Hospital for Sick Children, Toronto, Canada; 6 Department of Pharmaceutical Health Services Research, University of Maryland School of Pharmacy, Baltimore, MD, USA

## Abstract

**Background:**

Young adolescents’ and their parents’ experiences with Attention-Deficit/Hyperactivity Disorder (ADHD) and its treatment were explored to investigate beliefs and attitudes regarding use of stimulant medication, and their influence on treatment decisions.

**Methods:**

Using in-depth qualitative interviews, 12 adolescents with ADHD aged 12 – 15 years, and their parents described their experiences of ADHD and its treatment. Twenty four interviews, 12 with adolescents and 12 with their parents elicited detailed descriptions of beliefs about ADHD, attitudes about stimulant use and the circumstances surrounding treatment decisions. Verbatim transcripts were iteratively analyzed by a team of researchers following an interpretive interactionist framework.

**Results:**

Young people offered three themes describing ADHD: 1) personality trait, 2) physical condition or disorder, and 3) minor issue or concern. Regarding medication use, youth described 1) benefits, 2) changes in sense of self, 3) adverse effects, and 4) desire to discontinue use. Parents’ beliefs were more homogeneous than youth beliefs, describing ADHD as a disorder requiring treatment. Most parents noted benefits from stimulant use. Themes were 1) medication as a last resort, 2) allowing the child to reach his or her potential; and 3) concerns about adverse and long-term effects. Families described how responsibility for treatment decisions is transferred from parent to adolescent over time.

**Conclusions:**

Young adolescents can have different beliefs about ADHD and attitudes about medication use from their parents. These beliefs and attitudes influence treatment adherence. Incorporating input from young adolescents when making clinical decisions could potentially improve continuity of treatment for youth with ADHD.

## Background

Psychostimulant medications are highly effective for controlling the developmentally excessive inattention, overactivity, and impulsivity characteristic of Attention-Deficit/Hyperactivity Disorder (ADHD), as well as many of the associated disruptive behaviours, academic and social impairments [1]. If taken regularly, psychostimulants continue to be effective for two to five years, although clear documentation of long-term benefit remains elusive [2-4]. Despite use of stimulants, teenagers with ADHD remain at high risk for poor outcomes of academic and social underachievement, substance use, and frequent motor vehicle accidents [5]. Since rates of stimulant use decline as children become adolescents [6], poor medication adherence may be an important factor mediating poor outcomes.

Parents are the primary decision makers for young children, and as such, previous research has largely examined use of medication from their perspective. Parents describe the decision to use medications to treat their child’s ADHD behaviors as a difficult one, and they are more likely to start medications if they understand ADHD as a neurobiological condition, and that stimulant medications are safe [7-10]. Not surprisingly, parents who believe medication is unacceptable are unlikely to accept recommendations for use [11,12].

According to college students and older adolescents, the youth’s personal role in deciding about medication becomes increasingly important during adolescence [13,14]. Surveys with adolescents document that medication use is associated with the youth’s willingness to accept treatment and lessened awareness or concern about the stigma surrounding ADHD and medication treatment [15]. Interviews with teens with ADHD who use stimulants have shown that youth perceive benefits of treatment, although these are usually accompanied by negative side effects [14,16,17]. However, large gaps remain in what is known about young people’s experiences and how these influence medication use.

Many clinicians note that poor family functioning or parent–child conflict appears to play a role in discontinuation of medication. Qualitative studies where children and youth are interviewed provide a range of descriptions of how young people experience ADHD. Some studies report that children describe primarily negative experiences from having ADHD, [18-20] whereas elsewhere youth describe positive attributes of having ADHD or describe themselves as no different from their peers [13,16] . Children rate themselves as having fewer symptoms than their parents rate them [21] and may see themselves as functioning well compared with how others see them [22]. Parent and teen attitudes about treatment for ADHD can differ, [15] and these differences likely contribute to youth refusing to take medication as directed, [13] and ultimately discontinuation of stimulant treatment. To date, socio-cognitive models of behavior regarding use of medications to treat ADHD have focused primarily on the role of parental choice [10,23]. Costello et al. [24] applied the network episode model to decisions about child mental health care, with an emphasis on community, school and extended family influences. Recent conceptions of this model emphasize that the young person and his/her experience should be at the center of the model, [25] whereas in practice this rarely seems to be the case. Moreover, there is little evidence to inform clinicians about how and when to solicit input from young people instead of, or in addition to, parents in making treatment recommendations.

The current research explores the treatment experiences of young adolescents with ADHD and their parents in order to examine their beliefs about ADHD, attitudes regarding stimulant medication, and the parent–child decision-making process regarding the young person’s medication use during the developmental transition to early adolescence.

## Methods

The study methods and results are described in accordance with guidelines provided in the consolidated criteria for reporting qualitative research [26].

### Sample

Participants were twelve adolescents with a current or past diagnosis of ADHD and their parents, for a total of 24 interviews. The youth were chosen purposively to provide a sample that was six females and six males, and represented the age range from 12 to 15 years, with seven youth aged 12–13 years and five youth aged 14–15 years. In nine cases only the mother of the child participated in the parent interview; in the remaining three cases, both the mother and father of the adolescent participated. The children were chosen to represent a range of medication use histories. Seven youth were long-term users of stimulant medication (four continuously, range 6 months to 6 years, and three intermittently, range 4 to 6 years), two youth had recently started medication for the first time within the past 5 weeks, and three were not using medication at the time of the interview, one of these had had two brief trials and the other two had discontinued use one or more years previously. See Tables 1 and 2 for a description of the adolescents interviewed and their medication status.

**Table 1:** Description of adolescents: sample characteristics

	**N**	**%**
Sex, male	6	50
Age at interview		
12–13 yrs	7	58
14–15 yrs	5	42
Age at diagnosis		
7–8 yrs	5	42
9–10 yrs	4	33
11–12 yrs	2	17
14 yrs	1	8
ADHD, subtype		
C	6	50
IA	6	50
Comorbid		
LD	8	67
GAD/dysthymia	4	33
ODD	2	19

C: combined; GAD: generalized anxiety disorder; LD: learning disorder; IA: inattentive.

N: number of participants; ODD: oppositional defiant disorder; yrs: years; % percent.

**Table 2:** Description of adolescents: individual characteristics

**ID**	**Age (years) at**		**Medication status**				**Comorbid**		
	**Interview**	**Diag**	**On**	**On & off**	**D/C**	**ADHD subtype**	**LD**	**GAD/dysth**	**ODD**
F6	12	8		5y		C			
M1	12	11	6mo			IA	x	x	
F5	12	12	< 1mo			IA			
M2	13	7	6y			C			x
M3	13	8		6y		C	x		
M4	13	8		7y		IA	x	x	
F4	13	10	3y			IA	x		
F1	14	8			Brief trials	C	x		x
M5	14	9			On 6y, off 1y	C	x		
M6	14	9			On 4y, off 3y	C	x		
F3	14	14	< 1mo			IA	x	x	
F2	15	10	2y			IA		x	

C: combined; Diag: diagnosis at specialty clinic (some children were diagnosed by community MDs prior); D/C: discontinued psychostimulants; dysth: dysthymia; F: female; GAD: generalized anxiety disorder; LD: learning disorder; IA: inattentive; ID: case identification; M: male; mo: month; ODD: oppositional defiant disorder; > 1 yr: on psychostimulants more than 1 year; < 1 mo: on psychostimulants less than 1 month.

All youth had previously received a diagnostic assessment at a specialty clinic for children with attention, learning and behavior disorders. Inclusion criteria for the adolescents were DSM IV diagnosis of ADHD, recommendation of stimulant medication, age 12 to 15 years, one or both parents willing to participate, and ability to communicate in English. Youth who had other medical, developmental or psychiatric conditions that required ongoing medical treatment were excluded.

We approached youth who were identified by clinicians as likely to be willing to talk about their treatment experiences. Initially, one of the interviewers approached parents first followed by the young person; three youth preferred not to participate following their parent’s agreement, while two others agreed to schedule interviews and participate in the study. We changed strategies following these refusals and the interviewer approached the youth first, followed by the parent. None of the young people referred by their clinician refused when approached before the parent. Only one parent preferred not to participate. With this recruitment strategy there were two families who did not follow through with organizing an appointment following initial agreement to participate and a third family who was not home when the interviewers arrived, and could not easily reschedule with a follow up phone contact. Young people who were actively in treatment and using medication were more often referred by clinicians as willing to participate. As the data collection and analysis progressed, we purposely sought to speak with teens that had just started or were no longer using medication to obtain a range of subjective experiences. Clinicians were less able to suggest youth to approach who met these adjusted criteria. Both the adolescent and his/her parent or parents were approached to discuss participation in the study and interviews were scheduled at a subsequent time, allowing potential participants to change their mind. Formal consent with the parent and assent with the child were obtained immediately before the interviews according to the protocol approved by the Hospital for Sick Children Research Ethics Board, Toronto Ontario, Canada.

### Data collection

To ensure confidentiality, parents and youth were interviewed separately by teams of two researchers (EY, TG or AG) with experience in semi-structured interview techniques. Sampling and data collection continued until no new themes emerged regarding beliefs about ADHD and attitudes about medication use, consistent with the qualitative method underpinning this study.

The specific interview questions for the adolescents and their parents covered similar content areas, although the specific questions differed. The semi-structured interview guide for youth was adapted from one that was used in a focus group study examining subjective experiences of adolescents’ formal and informal supports for their difficulties with ADHD [23]. Questions and probes were selected and adjusted to accommodate the research objectives of the current study by the investigative team, which included members with extensive experience interviewing adolescents and their parents. For youth, we asked, “You have been told you have ADHD…what does that mean to you? In a typical day, how do you think having ADHD makes things different for you?”; “Tell me about your experiences with using medication for ADHD”, followed by probes to elicit the child’s perspective regarding use of medications in the contexts of school, home, with peers and with family. In contrast, the parent interviews were structured around questions related to the participant’s past and present experiences seeking help for the child’s ADHD, similar in design to previous research examining parent experiences [7-9]. For example, the interviewer asked parents, “Can you tell me something about your child’s problems with learning, attention and behavior? How did you decide that your child needed help?”; “Can you tell me about your experience with medication to help your child with learning, attention, and behavior?”. The aim of the interviews was to develop a “thick description” of all participants’ personal experiences; that is, a description that describes the participant’s experience in detail, including exploring the context surrounding each experience and the evolution of beliefs surrounding the experiences [27]. The interviews lasted between 60 and 90 minutes. Eight pairs of interviews were completed in the participants’ homes and four were conducted in the clinic at the family’s preference.

### Data analysis

Interviews and field notes were audio recorded and transcribed verbatim. Analysis was conducted following the interpretive interactionist framework set out by Denzin [27]. Under this framework, key elements are identified in individual interviews, compared across interviews to construct a larger picture, and then re-contextualized within the real world of the youth and parents.

In the initial phase of analysis, a team of four investigators representing several perspectives, including a pediatric psychiatrist, a sociologist, an education researcher, and a health researcher, independently read the transcripts and identified specific codes. Team members compared notes and developed a comprehensive code list through discussion following the initial interviews. The team met regularly and following each pair of interviews, the coding manual was revised; codes were refined, collapsed, or eliminated as needed. The interview questions and prompts for the semi-structured interview guides were also refined as the data analysis progressed. Consistent with the epistemology of qualitative research methods, the process of generating and refining codes continued until no new unique codes were identified.

Using the coding manual developed, all transcripts were compiled and organized using QSR International’s NVivo 9 qualitative data analysis software [28]. One investigator coded each transcript, and half the transcripts were recoded by a second investigator. Any discrepancies between codes were discussed with the whole team until 100% consensus was reached. The iterative process of organizing codes into categories was reflexive, using constant comparison between transcripts to identify common and recurrent themes.

Trustworthiness of the findings was established through accepted procedural strategies of *credibility, dependability, and confirmability* as recommended by Linclon and Guba [29]. Credibility was established through persistent observation, prolonged engagement in the field, and overall development of the project; dependability was established through an audit trail using fieldnotes, memo writing, and reflexive notes; and confirmability through multiple debriefings among the research team as they reviewed the analysis, interpretation, and representation of the data.

## Results

The interviews with the young adolescents and their parents provided rich narratives of their experiences with the diagnostic and the treatment process. In the first section, youth beliefs about ADHD and attitudes about medication treatment are summarized, with details provided in Tables 3 and 4. In the second section the parent beliefs and attitudes are summarized, with details provided in Table 4. In the final section, comments from adolescents and their parents describe the decision-making process for medication use.

**Table 3:** Adolescent beliefs about ADHD and attitudes about medication use


**Beliefs about ADHD**		
**Theme**	**Quote**	**Participant**
1) Personality traits	*…if I didn’t have ADHD, I don’t think I’d be me. …’cause if I didn’t have ADHD I’d bet you I’d be totally different.*	M2
	*I mean, it doesn’t make it that different to have ADHD, …I don’t see myself any different than anybody else.*	F5
	*It’s just a part of me kind of, it’s who I am.*	M1
2) Physical condition	*“Yeah, I have ADHD, what can I do?” Like I can’t control the way I was born, I can’t control the way my brain works.*	F5
	*I would just say like it’s a disorder - that just…anyway it’s just screwing up my life. ‘Cause like I’m treated kind of differently.*	F6
	*I think I still have [ADHD] but there’s only certain times it will come up. Sort of like the flu.*	M5
	*Well there’s kids who have stuff worse than me like diabetes and cerebral palsy.*	F6
	*ADHD, I have nothing, like there’s kids in Sick Kids’ Hospital that have cancer….*	F4
3) Being normal	*I don’t really care about most of this stuff. I just lead a normal day, a normal life.*	M3
	*Well for my mum it is [a problem] and for everyone else it is but with me, I don’t really care.*	M4
	*It’s not like something that’s like crossing my mind-ever.*	F2
**Attitudes about medication use**		
**Theme**	**Quote**	**Participant**
1) Benefits	*Without uh the medication, I wouldn’t be, I don’t even think like I’d be in school.*	M2
	*I definitely get my work done. It [the medication] sort of makes me feel like more of a normal student.*	F2
	*It’s an obstacle when I’m not on the pill.*	M1
	*When I took them? …I’d have…my confidence would boost up quite a bit?*	F4
	*My parents, they’re a lot happier, and my brother’s coming to me and my grammas’s not yelling at me anymore.*	F5
2) Effects on sense of self	*I just like notice when I don’t take the medication I’m happier, I’m more perky, I can get along with people.*	F6
	*I’m more of the quiet person who just sits there.*	F4
	*I don’t really feel like myself when I take it. …. You can tell the difference like between weekend me and school me.*	F2
	*I felt weird; I didn’t feel normal.*	M6
	*I didn’t really like the idea ‘cause I thought it was going to alter my brain…*	M1
	*I just didn’t want it to like affect me that much …- I thought it would change me.*	F1
	*I think…in a way, [taking medication] makes me feel normal? But in a way it doesn’t make me feel normal ‘cause it makes me feel a lot different from my friends.*	F2
3) Adverse effects	*It gave me headaches ‘cause they were too strong.*	M5
	*Made me like depressed and really moody all after school.*	M4
	*I’m sent to the office because I feel sick and I have to throw up. …I just couldn’t stomach any, eat anything.*	F6
		
4) Desire to discontinue	*If I weren’t taking the meds, I’d go to school happy and I think I’d have a much better day.*	M4
	*I’m not crazy about the medication. I wish I could stop if I could.*	F6
	*I guess I can just stop whenever … I learn to sort of work without, like maybe after high school I guess?*	F2
	*But I don’t usually take it during weekends or during the summer ….*	F4

**Table 4:** Parent beliefs about ADHD and attitudes about medication use

**Beliefs about ADHD**		
**Theme**	**Quote**	**Participant**
		**mother of**
Physical condition	*It’s really no different than somebody having something like cancer. They need to be treated.*	F1
	*… you have to wear glasses because you have poor eyesight, if you’re diabetic, you need insulin.*	F2
	*It’s almost as if there’s a cloud in the head, in his head that prevents his knowledge from coming out….*	M1
**Attitudes about medication use**		
**Theme**	**Quote**	**Participant**
		**mother of**
Last resort	*I always said that it’s a last resort, OK? And I mean other things I’ve tried, nothing’s y’know, nothing has helped.*	F1
	*I’ve been very judgmental about the whole medication thing before whenever …someone had mentioned their child had been on y’know Ritalin, I’d be like horrified.*	F4
	*But yet we’re here trying it one more time, ‘cause he’s not doing well without it.*	M4
Learning aid	*I explained to him that he is very bright and…., for him to uh reach his potential.*	M1
	*I presented the medication to him as being a learning tool, …it might make things easier for him.*	M6
	*I guess when you see that your child has potential and they’re not fulfilling their potential …you sort of realize [medication] is necessary.*	F2
Adverse effects	*…Like you’re giving this child medication, you’re losing…your child--the personality, the qualities. …“Oh what am I doing?”*	F5
	*I guess part of me worries about any possible long-term damage that it can cause?*	M1
	*I don’t know how it’s [medication]going to affect him in the future? I don’t know how it will affect his kids.*	M2

### Youth beliefs about ADHD

In the interviews, young people described how the diagnosis and symptoms of ADHD influenced their lives. Three dominant themes emerged: 1) ADHD as a personality trait, 2) ADHD as a physical condition or disorder, and 3) ADHD as “being normal”, warranting minimal concern. Several young people described characteristics from more than one theme category. See Table 3.

### ADHD as a personality trait: *If I didn’t have ADHD, I don’t think I’d be me*

For six youth, characteristics that might be attributed to having ADHD played a significant role in their understanding of who they are. These youth experienced symptoms and behaviours associated with ADHD as unique personality characteristics and a part of their self identity, rather than as impairments.

### ADHD as a disorder: *I can’t control the way my brain works*

In contrast to the view that ADHD is part of one’s personality, four youth understood it to be a disorder or illness that “happened” to them. These youth viewed ADHD as a physical condition or disorder that should be treated. Some youth who hold this ‘physical illness’ view of ADHD considered it to be less serious than other chronic medical conditions.

### ADHD as being normal: *I just lead a normal day, a normal life*

The third theme to emerge from participants’ narratives characterized ADHD as “normal”. In these discussions young people did not view their ADHD as impairing, nor was their diagnosis something that they think about frequently. Two youth had very little to say about how having ADHD affects their lives.

### Youth attitudes towards medication

For these young teens with ADHD, using stimulant medication is a complex experience. Major themes discussed in the interviews were: 1) benefits of medication; 2) medication and sense of self; 3) adverse effects; and 4) desire to discontinue medication. Most youth commented on all the themes, underlining how the experience of using medication is multifaceted. While some noticed benefits associated with stimulant treatment, they still had concerns about adverse effects, as well as the long-term implications of using medication. Even among those who noted benefit, some described effects on their sense of self and discussed eventual discontinuation. See Table 3.

### The benefits of medication: *It’s an obstacle when I’m not on the pill*

Youth noted positive experiences associated with taking medication, most commonly in school or while completing homework. Others experienced “secondary” benefits from medication, such as building their confidence in the classroom as well as in social relationships.

### Medication and sense of self: *The difference between weekend me and school me*

Approximately half the youth voiced concerns about the medication changing who they are, either a concern held when they had first started taking medication or as a current undesirable effect. Those who commented that psychostimulants changed their subjective experience of themselves reported feeling less sociable or outgoing when on the medication. Another experience described was that taking medication made a young person feel more like their peers, however, having to take medication also highlighted their apparent differences.

### Adverse effects: *I couldn’t stomach it*

The experience of adverse effects by youth reflected those known to be common with stimulant medication. Participants reported a number of unwanted side effects including difficulty sleeping, low appetite, mood swings, and stomach aches. Other complaints included difficulty swallowing the pills. The adverse effects experienced by young people had a number of consequences, such as trying different medications in search of one without side effects, adjusting the dose, as well as deciding to discontinue use.

### Discontinuing medication: *I wish I could stop*

At the time of the interviews, three young people were not currently using medication, while three others had previously discontinued usage and had restarted a new medication. Reasons given for stopping varied and included adverse effects, insufficient benefit, and the feeling that medication changed participants’ sense of self. Although the majority of participants were using stimulants at the time of the interview, a number of youth spoke of resisting use through non-compliance, wishing they could stop, or stopping at some unspecified time in the future. In some cases, however, young people were adherent and expressed satisfaction with their medication. They were able to negotiate how and when medication was used, for example only when attending school.

### Parent beliefs about ADHD

The interviews with parents revealed more homogeneity in their ideas and thoughts about ADHD than was noted among the adolescents. The majority of these parents viewed ADHD as a chronic medical illness or physical condition that required treatment or at the very least, special accommodations. See Table 4.

### Parent attitudes towards medication

Although the parents in our sample had children that had used medication for varying lengths of time, from just beginning to use medication to having used medication for several years, the transcripts revealed similar themes across interviews. When seeking help for their child, almost all parents: 1) viewed medication as a last resort; 2) understood medication to be something that helps their child reach his or her potential; 3) were concerned about adverse and long-term effects. Many, but not all, experienced benefits for their child, contributing to their decision to use medication. This decision process was complex and nuanced, and although all the parents in our sample chose to use medication for their child, several voiced uncertainty about that decision.

### Medication as a last resort: *I always said that it’s a last resort*

All parents commented that they were reluctant to begin medication and many described it as a last resort. For some parents, seeing the benefits after starting medication helped them understand that it was the “right” treatment for their child.

### Medication as aid to learning: *They’re not fulfilling their potential*

Parents saw the main benefit of stimulant treatment to be allowing their child to learn and function to the best of their abilities. Parents commented that when they decided to use medication, they hoped that it would allow their child to reach their full potential.

### Adverse and long-term effects: *You really don’t know what it’s going to do*

While most parents of youth currently taking medication could see improvements and benefits, they were also concerned about adverse effects and the potential for long-term complications.

### The decision-making process

Parents shared experiences about their choices to try medication and the ongoing decision-making process of continuing to use it or not. Over all parents described the initial decision, as largely parent driven, as the child was often too young to participate.

…*at that age, (grade 3) we told him this is what it was gonna be. (*Mother of M5)

Parents also described that as their child grew up, they relinquished control over the decisions around medication use to varying degrees.

*If he has work to do, he’ll usually choose to take the four hour one? He sort of knows himself how much time is required to do it*. (Mother of M1)

According to one mother, the doctor broached the subject of going off meds and her son jumped at the chance.

*I almost didn’t have a say any longer.* (Mother of M5)

The transfer in responsibility described by parents was reflected in youth narratives as well, with the young person choosing whether to take medication some or all the time:

*… mom gave me a choice.* (F4)

*…the decision is up to me.* (M6)

*I have told mom I will go back on [medication].* (F1)

Several themes accompanied the transfer of responsibility to the young person, among them how the parent perceived the young person’s maturity, *“I think she realizes that she’s successful in school when she takes it” (*Mother of F2); a perceived need for the youth to be more in charge of his /her life, “*he’s going to be out in the world soon, I can’t be mothering him.”* (Mother of M5); and, the youth’s increased ability to express him or her self, *“this year’s been different for a couple of reasons. She’s older…started to voice her opinion.”(*Mother of F6).

In some cases, the decision to discontinue medication followed a series of different medication trials with less than optimal results. For adolescents who had stopped medication,

*“it didn’t seem to be helping him sufficiently in school” (*Mother of M6).

Tension in the relationship between child and parent regarding medication use was clearly present on more than one occasion, a theme often expressed in the narratives of both the young person and the parent.

*Medication this, medication that…Well sometimes medication doesn’t help!* (F6)

*I feel so strongly about it because I don’t see what else is gonna work… these are such crucial years.* (Mother of F6)

Participant M4 stated that it is annoying to take medication, because *“my mom tries to force me to take it.”* In our interview with M4’s mother, she described a talk she had with her son:

…we’ve promised your teachers, we’ve wasted (the clinician's) time and y’know we need to try this…and then I make him do it.

The young people currently in conflict with their parents about using medication emphasized the lack of benefit, the unpleasant adverse effects, and their happiness when not taking it.

*If I weren’t taking the meds, I’d go to school happy and I think I’d have a much better day.* (M4)

*I just like notice when I don’t take the medication I’m happier, I’m more perky. I can get along with people.* (F6)

*But I’m on this new stuff now? They’re putting me on such a low dose that I don’t even think it’s going to make a difference.* (M4)

*I think the medication…it’s kind of impairing my learning because a lot of time I’m sent to the office because I feel sick….* (F6)

Youth who had already discontinued using stimulants echoed similar themes.

*And I was kind of like, I would kind of like lose energy, I was kind of tired. I felt kind of isolated. I felt kind of overwhelmed.* (M6)

In contrast, a number of young people recognized the benefits, sometimes tolerating negative effects (i.e. being quieter, less social), and chose to remain on medication over time,

“*makes me feel more like a normal student”* (F2)*;*

*“there was a really big difference.* (F4);

“*like putting on a seat belt… like the one day I don’t take it and I’m having a quiz …-I just don’t need that day.* (F2)

Not surprisingly, as the responsibility for decision-making about medication use shifted from parent to adolescent, the youth’s subjective experience determined whether or not they continued to take it on a regular basis.

## Discussion

Our interviews with young adolescents and their parents revealed a more diverse range of beliefs and attitudes about ADHD among young people than among their parents. These differences underscore the importance of the young person’s perspective in making decisions about their treatment for ADHD. Several youth viewed their ADHD as a chronic physical disorder, a belief shared almost uniformly by the parents we interviewed. While parents who held this belief generally expressed support for medication use, this was not the case with the young people. One youth who expressed this belief (M5, see Tables 2 and 3) no longer took medication while another described arguing with her mother about continuing it (F5). Another belief expressed among adolescents was that ADHD behaviors are part of who they are, i.e., their self-identity, or are a minor inconvenience, not affecting their day-to-day experience. The diversity of comments elicited in the current study regarding the personal meaning of ADHD mirrors the diversity noted across and within qualitative studies of adolescents [13,16,17,19,20]. Few of the young people interviewed in this study emphasized negative meanings. The positive comments heard here contrast with earlier reports that children with ADHD frequently referred to themselves as “bad” and may have difficulty describing positive attributes of having ADHD [18,20]. On the other hand, these positive beliefs about ADHD have also been noted elsewhere [16] and echo the themes of positive group identity as discussed by Gajaria et al. [30] in their analysis of public Facebook groups used by youth with ADHD. In addition, these themes are reminiscent of the phenomenon of positive illusory bias, where youth with ADHD do not see themselves as functioning as poorly as others see them [22]. Overall, however, such diversity of beliefs and attitudes within and across samples of adolescents with ADHD is to be expected, given the diversity of interview contexts and investigator points of view, range of research questions, non-standardized interview guides, methods of recruitment, and generally small samples of volunteer participants ranging in age from childhood to late adolescence.

The adolescents’ attitudes toward medication use reflected their beliefs about ADHD. For example, worries expressed about having a changed personality with medication maps onto the understanding of ADHD behaviors as a positive aspect of self. On the other hand, descriptions of benefit with medication use may reflect the theme of ADHD as a chronic disorder that requires intervention. Young people who experienced benefit, also described potentially unwanted effects, (F2, F4, see Table 3) but tolerated them, for example by using medications on school days, but not on weekends. Among the children currently in conflict with parents over using medication, the benefits of medication were not great enough to outweigh the adverse effects (M4, F6 see Table 3).

A unique aspect of our study was the concurrent interviewing of young people and their parents, thereby avoiding the potential for parents and children to directly influence each other’s responses. Overall these parents held a relatively homogenous conceptualization of their children’s difficulties and the role of medication, in contrast to the diverse views expressed by their children. Consistent with previous studies of parental views, these parents viewed ADHD as a long-term problem, with the choice for medication treatment a “last resort”; and their concerns centered on adverse and long-term physical effects [7-11]. As a group these parents anticipated that medication use would help their children reach their full potential, a theme more often voiced by parents who choose medication than those who avoid it [10,11].

We heard from both parents and adolescents that as the young persons grew older, they were given greater responsibility by their parents and health care providers, and their opinions increasingly dominated decisions about medication use. Not surprisingly, the transfer of decision-making from parent to youth occurred more easily for those parent–child pairs where the adolescent and the parent agreed about whether to use medication or not. Our interviews captured two parent-youth pairs who were in the midst of active conflict regarding use of medication and for whom transfer of responsibility was not an easy process. These examples provide a simultaneous parent–child lens on the increasing participation by young teenagers in their treatment decisions about ADHD.

The results of this study are exploratory and limitations include the small sample recruited through a specialty clinic where most families had a history of ongoing engagement in treatment. While our sample may be small in number, our goal is not to generalize results to large numbers of teens with ADHD but to investigate in-depth the phenomenology of beliefs and attitudes about ADHD and decisions about stimulant medication use, within the contextual frame of parent–child relationships and early adolescence. The experiences of our sample reflect primarily those of families engaged in ongoing treatment, and this is an important group for whom understanding the phenomenon of transferring medical decision-making from parent to youth during early adolescence is highly applicable. Indeed we purposefully sought out young people who had recently started medication or who were not taking medication at the time of the interview in order to hear a variety of beliefs and attitudes and to capture descriptions of crucial moments in the parent -child decision-making process. Parents in the current study describe the process of transferring responsibility as they see their child becoming more capable. In a study focused on medication treatment decisions among ADHD youth, Brinkman et al. [13] interviewed focus groups of adolescents, ages 13 to 18 years, recruited in primary care settings where the majority of stimulant medication is prescribed. They documented a range of participation in medication decisions with some young people having full autonomy [13].

While there is some controversy in the field about the concept of reaching saturation and when to stop recruitment [31], we easily reached the point where there were no new emerging themes on the primary questions examining the beliefs and attitudes of young people and those of their parents, as well as obtaining subjective accounts of decision-making shifts from parent to adolescent.

Overall, the study findings underline how the perspectives of young people with ADHD may differ from their parents in ways that have important implications for ongoing adherence to stimulant treatment. These preliminary results confirm that divergent opinions between a youth and his or her parents can appear early in the teen years. While clinicians who work with young people know that adolescents should play an active role in their own health care as they get older, the majority of prescriptions for ADHD medications are written by primary care practitioners [32], who are often busy and may or may not have time to speak directly with the young person themselves. Even for pediatric specialists, when and how the transition from parent decision-making to joint parent and teen decision-making should occur is not always clear. Many children remain quiet or passive during treatment decision conversations and apparently acquiesce to what the adults plan [13]. However, our results suggest that even when children accept the treatment plan, their point of view may differ from that of their parent. For some families, when young people do not feel their opinions regarding treatment are heard, it can become a source of parent–child conflict, or result in poor treatment adherence. As clinicians who work with adolescents know from experience, the best approach often includes a conversation with the young person on his or her own, soliciting the young person’s opinions and preferences, and offering opportunities to ask questions. A subsequent joint conversation with child and parent together is often required to complete the planning process.

## Conclusions

In summary, little evidence exists about the process of health-care decision making for young adolescents and their parents. In this preliminary study, we describe how beliefs and attitudes about ADHD in children ages 12 to 15 sometimes differ from those of their parents. Not surprisingly, the young person’s beliefs and attitudes have increasing impact on medication use as decision-making shifts from parent to youth. Clinicians can facilitate improved medication adherence by working both with the young adolescent as well as their parent when developing the treatment plan.

## Competing interests

The authors declare that they have no competing interests.

## Authors’ contributions

AC initiated study concept, assisted in study design and analyses and was the primary author of the manuscript. EY assisted with study design, interviewed participants, was primary data analyst, and assisted in writing the manuscript. TV participated in study design and analyses, reviewed and revised the manuscript. TG interviewed participants, assisted with study design and analyses, reviewed and revised the manuscript. SD advised in study design and analyses, reviewed and revised the manuscript. All authors read and approved the final manuscript.

## Pre-publication history

The pre-publication history for this paper can be accessed here:

http://www.biomedcentral.com/1471-244X/14/110/prepub
